# Identification of fragments from Autographa Californica polyhedrin protein essential for self-aggregation and exogenous protein incorporation

**DOI:** 10.1186/s12858-015-0034-9

**Published:** 2015-02-04

**Authors:** Alicia Sampieri, Agustín Luz-Madrigal, Jesus Zepeda, Luis Vaca

**Affiliations:** Instituto de Fisiología Celular, Universidad Nacional Autónoma de México, Ciudad Universitaria, México, DF 04510 México; Department of Biology and Center for Tissue Regeneration and Engineering, University of Dayton (TREND), Dayton, OH USA; Department of Biology, Miami University, Oxford, OH USA

**Keywords:** Autographa Californica, Polyhedrin, Polyhedra, Baculovirus

## Abstract

**Background:**

Baculoviruses are widely used for the production of recombinant proteins, biopesticides and as gene delivery systems. One of the viral forms called polyhedra has been recently exploited as a scaffold system to incorporate or encapsulate foreign proteins or peptide fragments. However, an efficient strategy for foreign protein incorporation has not been thoroughly studied.

**Results:**

Based on the crystal structure of polyhedrin, we conducted an *in silico* analysis of the baculovirus *Autographa californica nucleopolyhedrovirus* (AcMNPV) polyhedrin protein to select the minimum fragments of polyhedrin that could be incorporated into polyhedra. Using confocal and transmission electron microscopy we analyzed the expression and cellular localization of the different polyhedrin fragments fused to the green fluorescent protein (EGFP) used as reporter. The amino fragment 1–110 contains two repeats formed each of two β sheets followed by a α helix (amino acids 1–58 and 58–110) that are important for the formation and stability of polyhedra. These fragments 1–58, 58–110 and 1–110 could be incorporated into polyhedra. However, only fragments 1–110 and 58–110 can self-aggregate.

**Conclusions:**

These results demonstrate that 58–110 is the minimum fragment that contributes to the assembly of the recombinant polyhedra via self-aggregation. This is the minimum sequence that can be used to efficiently incorporate foreign proteins into polyhedra.

## Background

The development of eukaryotic systems for the expression of recombinant proteins has been a major goal in biotechnology due to its widespread utility in medicine, veterinary medicine, and agriculture, among other related areas [1].

The use of insect viruses to produce and to obtain different recombinant proteins has grown in recent decades [2,3]. Three of these eukaryotic systems are expressed in insect cells and are currently in use. Two of them are based on the DNA baculoviruses *Autographa californica nucleopolyhedrovirus* (AcMNPV), and *Bombyx mori* nucleopolyhedrovirus (BmNPV). The third uses the RNA virus *Bombyx mori* cytoplasmic polyhedrosis virus (BmCPV) cypovirus. In nature, the viruses of these 2 families are protected from adverse environmental conditions as they are occluded into crystalline lattices called polyhedra or occlusion bodies, derived mainly from a single viral protein called polyhedrin [4]. The occlusion is an adaptation that allows baculoviruses to remain in a dormant but infective state in the environment for decades [5].

Polyhedrin is one of the most abundant proteins in a baculovirus-infected cell, since its expression is driven by a very strong promoter [6]. Because polyhedrin is not necessary for the propagation of the virus, the DNA sequence of the protein can be replaced with some other sequence of interest [7]. This in turn, has allowed the polyhedrin promoter to be used as an expression strategy for obtaining high yields of recombinant proteins.

Since BmNPV and BmCPV polyhedra are particles of about 1 μM in diameter and can be easily purified by centrifugation, they represent good candidates to express recombinant proteins. Using this strategy, Je et al. incorporated the green fluorescent protein (GFP) into the AcMNPV polyhedra by fusing it to the carboxyl terminus from the polyhedrin gene [8]. However, the expression of the recombinant protein did not form polyhedra [8]. Only the combined expression of both the wild type (WT) and the recombinant polyhedrin (GFP-polyhedrin) proteins resulted in the formation of polyhedra [8]. This result shows that fusing proteins to polyhedrin prevent the formation of polyhedra, but WT polyhedrin can rescue this phenotype. Nevertheless, these results highlight the little we understand about how polyhedra particles are formed in the nucleus of baculovirus infected cells.

The polyhedrins of baculoviruses and cypoviruses do not share a similar amino acid sequence [9]. However, the crystal structures of both polyhedra are indistinguishable between the two families in terms of their size and symmetry [10,11]. Thus, these conserved properties suggest that the crystal structure of polyhedra has been retained in nature for the specific purpose of preserving the viruses, and that such crystalline structure can be obtained using proteins with different amino acids compositions.

It has already been shown that the crystal structure from both the AcMNPV and the BmCPV polyhedra is an arrangement of polyhedrin trimers, which are interconnected through their amino N-terminal helices [10]. These interactions provide significant stability to the polyhedra, since the trimer is the base of the crystal core [11]. The identification of the properties of the crystallography structure has allowed investigators to determine the interacting amino acids in the crystal formation and to identify which of them are necessary for configuring the polyhedra core structure [11,10].

Despite the similarities in crystal symmetries and identical unit cell dimensions, structures of baculovirus and cypovirus polyhedrins are different at the atomic level. Both structures have a β-sandwich core domain, with projecting C- and N-terminal helices, but the topologies are dissimilar and the helices interact differently [12].

Based on these findings, Ijiri et al. incorporated several foreign proteins into BmCPV polyhedra by fusing them to the first 30 amino acids of polyhedrin, which contains an α helix known as H1 [13]. Because this fragment projects towards the outside of the protein, it forms independently as the molecule folds; it interacts with other molecules of polyhedrin and it is incorporated into polyhedra crystal structure. Thus, the co-expression of H1 with the WT polyhedrin is now widely used as a tag to incorporate foreign proteins into BmCPV polyhedra [13-15].

More recently, recombinant polyhedra in BmNPV have been obtained by co-expressing the foreign proteins fused to the first 110 amino acid N-terminal fragment in combination with the complete WT polyhedrin [16,17]. The foreign proteins were then purified from polyhedra [16,17].

**Figure 1:**
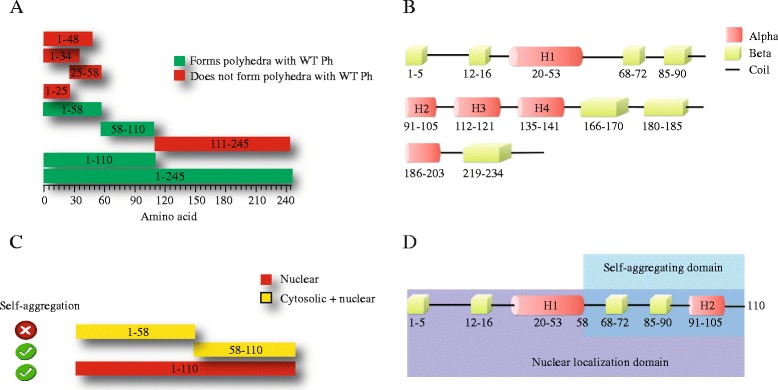
***Identification of different motifs for nuclear localization, self-aggregation and incorporation into polyhedra in polyhedrin.*** **A**, diagram illustrating the different fragments from the polyhedrin protein tested in this study. In red as depicted the fragments that are not incorporated into the polyhedra crystal (when co-infected with a virus carrying a copy of WT polyhedrin). Inside the rectangles are indicated the amino acid numbers for the different fragments. **B**, analysis of the secondary structure of wild type polyhedrin, obtained from the crystallographic structure (2WUY.pdb, http://www.rcsb.org/pdb/explore.do?structureId=2wuy)). Red barrel depict α helices and green cubes β sheets, while coils are depicted as straight lines. **C**, identification of the self-aggregating fragments from polyhedrin and its cellular localization. Red indicates only nuclear and yellow nuclear and cytosolic. **D**, diagram indicating the self-aggregating domain and nuclear localization domain in the N-terminal region from polyhedrin. Domains are shaded in color for easier identification.

Because AcMNPV is one of the most widely used systems to express recombinant proteins, and given the fact that less is known about what fragments in the polyhedrin protein are sufficient to incorporate foreign proteins into polyhedra, we aimed the present study at determining the minimal fragment that can be used to incorporate foreign proteins into AcMNPV polyhedra. We first analyzed the amino terminal of AcMNPV polyhedrin considering the known crystal structure of the protein (Figure 1B and [10]). Based on the structural features, we produced different fragments to explore which ones can be incorporated into the polyhedra crystal.

These findings unravel the role of the amino region 58–110 from polyhedrin in the assembly of AcMNPV polyhedra, while providing the bases for a system to incorporate efficiently high levels of recombinant proteins into the polyhedra crystal.

## Methods

### Insect cell line and baculoviruses

The *Spodoptera frugiperda* cell line, Sf9, was maintained at 27°C in Grace medium (Invitrogen, USA) supplemented with 10% heat-inactivated fetal bovine serum (56°C, 30 min) (Wisent, Inc., Canada), 1X Yeastolate (Invitrogen, USA), 1X Lactalbumin (SIGMA, USA), and 1X Antibiotic-antimycotic (Invitrogen, USA) according to standard methods. For suspension cultures, pluronic acid F-68 at a final concentration of 0.1% was added and the cells were sub-cultured every 2 to 3 days. The Bac-N-blue system (Invitrogen, USA) was used for the construction of the recombinant baculovirus (see below). The WT and recombinant AcMNPVs used in the present study were propagated in Sf9 cells.

### Generation of the recombinant baculovirus

The complete polyhedrin gene was obtained by PCR amplification using the Baculovirus forward and reverse PCR primers (Invitrogen, USA) and cloned into pEGFP-C2 (Clontech, USA). The recombinant baculoviruses with the different fragments of polyhedrin were constructed by digestion with restriction enzymes or by PCR amplification of the constructions. These PCR fragments were cloned to pEGFP-N1, −N2 or -N3 (Clontech, USA) as needed to obtain the fusion proteins with an open reading frame. The recombinant plasmid vectors were confirmed by restriction endonuclease analysis and sequencing. All the fusion genes of EGFP-polyhedrin were then sub-cloned into the pBlueBac4 plasmid (Invitrogen, USA), which was used with the Bac-N-blue transfection kit (Invitrogen, USA) to obtain the recombinant baculoviruses. The recombinant baculoviruses were purified and then amplified to obtain high titer virus stocks. The baculovirus titer was obtained and expressed as plaque forming units (pfu) per milliliter according to standard protocols provided by the manufacturer (Invitrogen, USA).

### Production and purification of recombinant polyhedra

Sf9 cells co-infected with the recombinant and WT viruses were collected by centrifugation at 96 hrs post-infection, resuspended in phosphate buffered saline (PBS; 20 mmol/L NaH_2_PO_4_, 20 nmol/L Na_2_HPO_4_, 150 mmol/L NaCl, pH 7.2) (Sigma, USA), and fragmented ultrasonically three times for 30 s each (Braun Biotech International, Germany), followed by centrifugation at 12,000 *g* at 4°C for 10 min. The pellets were then washed 2X with PBS and then finally resuspended in PBS buffer.

### Confocal microscopy 3D reconstructions and electron microscopy scanning

The recombinant purified polyhedra of infected insect cells were allowed to adhere to each of the wells of a LabTek II Chamber Slide (NalgeNunc Int, USA). The polyhedra or cells were washed three times with PBS and fixed with mounting medium (DakoCytomation, USA). The infected insect cells were incubated with DAPI (4,6-Diamidino-2-Phenylindole, Dihydrochloride), Molecular Probes, USA) at a dilution 1:1000 for 5 min before fixation with DAKO Cytomation fluorescence mounting media (DakoCytomation, Denmark). Images were collected with an Olympus FV1000 confocal microscope and analyzed using Fluoview 10-ASW-2.1 software (Olympus, Japan). Image acquisition using transmission electron microscopy (TEM) of infected cells was conducted according to established protocols. Briefly, the cells were washed with 0.08 M cacodylate buffer (Sigma St. Louis, MO) and fixed for 10 min with 0.6% glutaraldehyde (Sigma St. Louis, MO) and 0.4% paraformaldehyde in 0.08 M cacodylate buffer, pH 7.4. Post-fixation was made with 1% osmium tetroxide (Fluka, St. Louis, MO) in cacodylate buffer Thin sections were counterstained with uranyl acetate for 10 minutes and with lead citrate for 2.5 minutes. Observations were made in a Jeol 1010 electron microscope (Jeol USA, Peabody, MA).

### Flow cytometry studies

**Figure 2:**
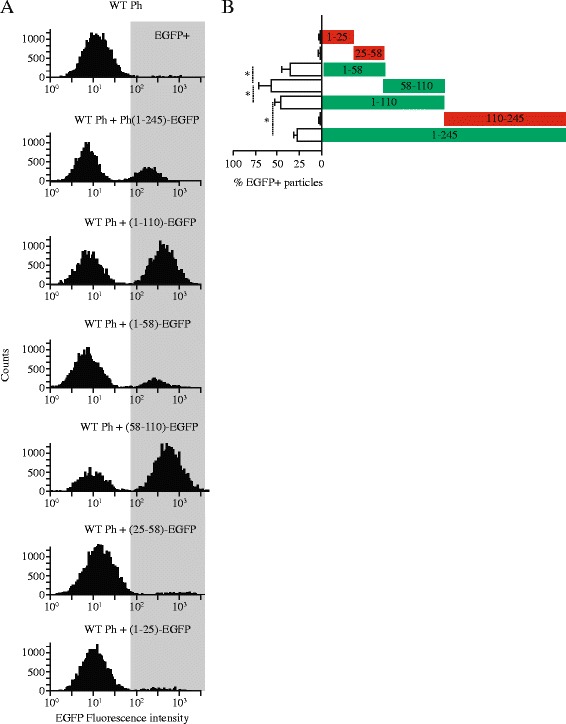
***Different efficiencies of incorporation into the polyhedra crystals obtained with the different fragments studied*** **. A**, flow cytometry studies of purified polyhedra crystals obtained with the different fragments indicated in the figure. In all cases a MOI of 1 was used for each EGFP containing polyhedrin fragment and a MOI of 3 for wild type polyhedrin. Crystals purified from Sf9 cells subjected to sonication (Methods). Fluorescence intensity collected in the 525 nm emission channel (Methods). In all cases 10,000 events were collected for each polyhedra. Autofluorescence (fluorescence background) was determined using wild type polyhedra (without EGFP), as indicated in the first panel at the top. Using this background level we identified the EGFP positive fluorescence (EGFP+, indicated by the gray rectangle). **B**, percentage of EGFP+ events (individual polyhedra crystals) obtained from the histograms shown in A. Notice polyhedra crystals produced with fragment PH_(58–110)_­EGFP produced the highest EGFP intensity values, followed by PH_(1–58)_­EGFP and PH_(1–110)_­EGFP. Notice that PH_(1–25)_­EGFP, PH_(25–58)_­EGFP and PH_(110–245)_­EGFP did not produce fluorescent polyhedra. Flow cytometry data is in agreement with the results obtained with confocal microscopy (Figure 7).

The polyhedra crystals containing the different fragments of polyhedrin fused to EGFP were purified as indicated above. Purified crystals were subjected to confocal microscopy and TEM to validate its purity. In all cases basically no cellular debris was observed. Polyhedra crystals were introduced into the sorting chamber of a fluorescence-activated cell sorting (FACS) apparatus (FACSCalibur, BD Biosciences). Event counting was terminated at 10,000 events as previously described [18]. Fluorescence signal was collected at 525 nm (EGFP emission peak) and plotted in logarithmic scale in histograms illustrated in Figure 2A. Counts or events reflect single polyhedra particles. These measurements were utilized to calculate the percentage of GFP positive crystals, using wild type polyhedra (without EGFP) to identify the autofluorescence (background) level.

### Nanoparticle Tracking Analysis (NTA)

**Figure 3:**
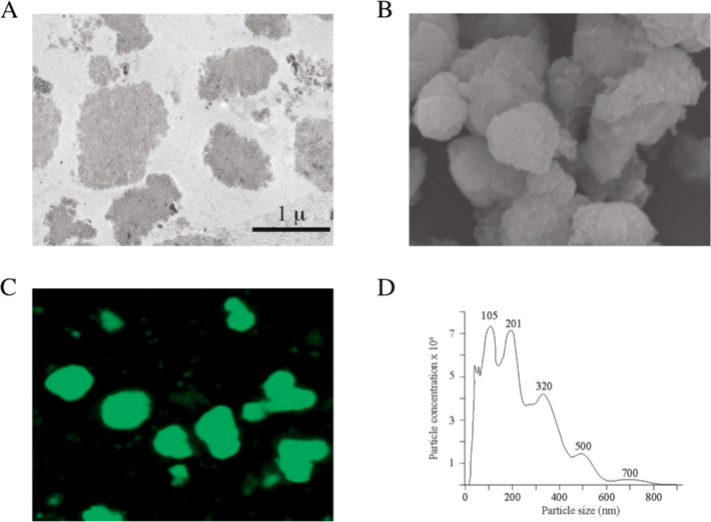
***Polyhedrin 58–110 and 1–110 produce electron dense nanoparticles.*** **A**, Representative transmission electron microscopy (TEM) of the segment of the cell nucleus from a Sf9 cell infected with a recombinant baculovirus expressing PH_(1–110)_­EGFP. The bar scale indicates 1 micron and applies to all panels in the figure. **B**, representative scanning electron microscopy of purified nanoparticles produced by the expression of PH_(1–110)_­EGFP. **C**, confocal microscopy of purified nanoparticles produced by the expression of PH_(1–110)_­EGFP. **D**, identification of main nanoparticle sizes in solution using Nanoparticle Tracking Analysis (NTA). Notice that all nanoparticles identified are multiples of the smallest size identified with NTA of approximately 100 nm. Numbers next to each peak identify the mean peak nanoparticle size value in nanometers (nm). All nanoparticles were purified from Sf9 lysates and isolated by low speed centrifugation, as indicated in material and methods. Identical nanoparticles were observed when using PH_(58–110)_­EGFP (data not shown).

NTA is becoming a standard method for submicron (nanoparticle) particle analysis [19]. This technique combines laser light scattering microscopy with a charge-coupled device (CCD) camera, enabling the visualization and tracking of nanoparticles in solution. Nanoparticle sizing is derived from the Stokes-Einstein equation by studying the Brownian motion of the nanoparticles and the way light is scattered during motion [20]. Thus, this method is particularly useful for studying nanoparticles in suspension and can identify nanoparticle aggregates [20]. For these experiments we have utilized the NanoSight NTA system from Malvern (Amesbury, United Kingdom). Purified nanoparticles from PH_(1–110)_­EGFP and PH_(58–110)_­EGFP were introduced in the system at two different concentrations. Particles sizes and particle concentration was obtained from direct measurements with NanoSight. Data represents the analysis from millions of events and is given by particle sizes per milliliter (Figure 3D).

### Polyhedrin modeling

The crystal structure of the wild type AcMNPV polyhedrin (2WUY.pdb, http://www.rcsb.org/pdb/explore.do?structureId=2wuy)) was utilized in the identification of the polyhedrin secondary structure, which directed the generation of the different polyhedrin fragments reported in this study.

## Results

### Recombinant baculoviruses expressing polyhedrin fused to EGFP

In order to visualize the expression and localization of polyhedrin and polyhedra in the infected cells, we developed several recombinant baculoviruses containing different fragments from the polyhedrin gene fused to the enhanced green fluorescent protein (EGFP). The baculovirus were designated according to the amino acid fragment of polyhedrin: WT polyhedrin (PH_(1–245)_-EGFP), and the different fragments of polyhedrin: amino acids 1–25 (PH_(1–25)_­EGFP), 1–34 (PH_(1–34)_­EGFP), 1–48 (PH_(1–48)_­EGFP), 1–58 (PH_(1–58)_­EGFP), 17–58 (PH_(17–58)_­EGFP), 58–110 (PH_(58–110)_­EGFP), 1–110 (PH_(1–110)_­EGFP), and 111–245 (PH_(111–245)_­EGFP). All recombinant genes were under the control of the polyhedrin promoter in the recombinant baculoviruses. All polyhedrin fragments were cloned at the N-terminal of the EGFP except for PH_(1–245)_­EGFP which was cloned at both the N- and C-terminal of the EGFP. Because we obtained indistinguishable results with both constructs, we will describe here only PH_(1–245)_-EGFP. In order to obtain polyhedra, a second recombinant baculovirus carrying the wild type copy of polyhedrin was utilized in co-infections with all baculoviruses carrying fragments from the polyhedrin gene (listed above).

### Recombinant PH_(1–245)_-EGFP was expressed in the cytoplasm of Sf9 infected cells but it retains the ability to form aggregates

**Figure 4:**
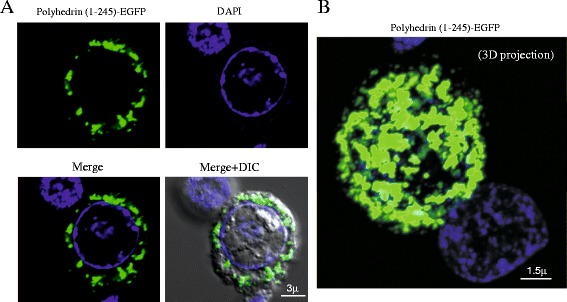
***Polyhedrin fused to EGFP forms cytosolic aggregates.*** **A**, confocal microscopy visualization of the cellular localization of full-length polyhedrin fused to EGFP (PH_(1–245)_-EGFP). Panel on the left side shows EGFP fluorescence, and panel on the right DAPI staining to illustrate the localization of the nucleus. Lower panel shows the merge (EGFP + DAPI) and to the right the merge + differential interference contrast (DIC). **B**, confocal tridimensional projection of a cell expressing PH_(1–245)_-EGFP. Notice that DAPI labeling is covered by the PH_(1–245)_-EGFP fluorescence, because PH_(1–245)_-EGFP is expressed in the cytosol. Notice the formation of aggregates by PH_(1–245)_-EGFP.

The distribution of PH_(1–245)_-EGFP was analyzed by confocal microscopy in Sf9 infected cells. Figure 4 shows that the recombinant PH_(1–245)_-EGFP did not form polyhedra. Similar results were observed in cells infected with the same polyhedrin fragment cloned in the N-terminus of EGFP (data not shown). In both cases EGFP was observed as aggregates (see 3-D confocal projection) localized at the cytoplasm of the cells. EGFP never co-localize with DAPI counterstained nuclei, demonstrating the cytosolic localization of PH_(1–245)_-EGFP (Figure 4). These findings suggested that the EGFP disrupts the transit of the full-length polyhedrin to the nucleus, preventing the assembly of polyhedra. Nevertheless, this construct can form aggregates on its own (without wild type polyhedrin).

### WT polyhedrin is required for the formation of polyhedra

**Figure 5:**
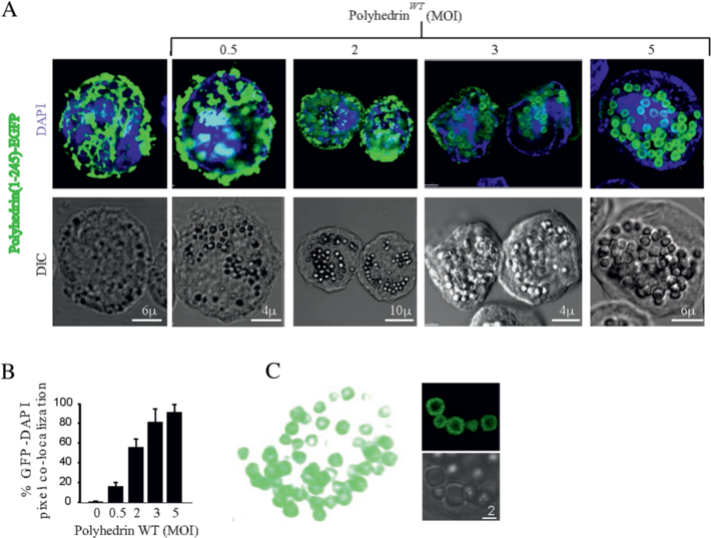
***Titration of the amount of WT polyhedrin required to integrate*** **PH**_**(1–245)**_ **-EGFP** ***into the polyhedra crystal.*** **A**, upper panels show the fluorescence of PH_(1–245)_-EGFP and DAPI, while lower panels show differential interference contrast (DIC) in cells co-infected with baculoviruses carrying PH_(1–245)_-EGFP (multiplicity of infection , MOI =1) and a second baculovirus carrying a wild type copy of polyhedrin. The second baculovirus was utilized at increasing MOIs of 0.5, 1, 2, 3, 4 and 5 (only 4 MOIs shown for illustration purposes). Notice that at a MOI of 3 (and above), all PH_(1–245)_-EGFP was contained inside the polyhedra crystals. **B**, percentage of pixel co-localization between EGFP and DAPI, to determine the amount of PH_(1–245)_-EGFP present inside the nucleus. Notice that with a MOI of 3, all PH_(1–245)_-EGFP localizes in the nucleus (no differences were observed between MOIs of 3 and 5). **C**, polyhedra containing PH_(1–245)_-EGFP were purified by centrifugation from Sf9 insect cells subjected to sonication. Notice that all polyhedra were fluorescent, indicating that PH_(1–245)_-EGFP was present.

In order to obtain recombinant polyhedra with EGFP incorporated into the crystals, we performed a series of co-infections using both WT and recombinant baculoviruses carrying PH_(1–245)_-EGFP. In all these experiments two viruses were used, one carrying a WT copy of full-length polyhedrin, and a second virus carrying the corresponding polyhedrin fragment fused to EGFP. After performing a WT baculovirus- PH_(1–245)_-EGFP baculovirus co-infection titration, we concluded that the ratio of WT polyhedrin to recombinant PH_(1–245)_-EGFP was critical for the formation of polyhedra (Figure 5). Figure 5A shows tridimensional confocal reconstructions of Sf9 cells co-expressing PH_(1–245)_-EGFP and the WT polyhedrin, obtained by maintaining the multiplicity of infection (MOI) of the baculovirus PH_(1–245)_-EGFP at 1 and by increasing the MOI of baculovirus carrying the WT copy of polyhedrin from 0.5, 1, 2, 3 and 5 MOI. In Figure 5B, a pixel co-localization analysis of EGFP/DAPI shows that polyhedra formation in the cell nucleus occurred when a ratio of 3 or more MOI of WT polyhedrin to 1 MOI of PH_(1–245)_-EGFP was used. This result revealed that WT polyhedrin had to be co-expressed in order for recombinant polyhedrin fragments carrying EGFP to locate and assemble into polyhedra in the nuclei of the cells. The requirement of WT polyhedrin for the formation of recombinant polyhedra has been previously demonstrated for other baculoviruses [17,21,22]. Interestingly, the ratio of WT and recombinant polyhedrin copy has not been determined until now. Our study highlights the need to use an adequate ratio (3:1) of WT versus recombinant polyhedrin in order to secure the incorporation of all recombinant copies into polyhedra. Altering this ratio results in an excess of soluble recombinant polyhedrin copies suspended in the cell cytosol. The 3:1 ratio is an interesting number, given the fact that the core of the polyhedra crystal is a trimer. These results suggest that every wild type trimer may contain a copy of the PH_(1–245)_-EGFP. Increasing the ratio of PH_(1–245)_-EGFP results in soluble protein, suggesting that all possible sites have been occupied in the polyhedra and the excess PH_(1–245)_-EGFP is discarded from the crystal structure.

### Polyhedra-like formation in Sf9 cells infected with PH_(1–110)_­EGFP baculovirus

**Figure 6:**
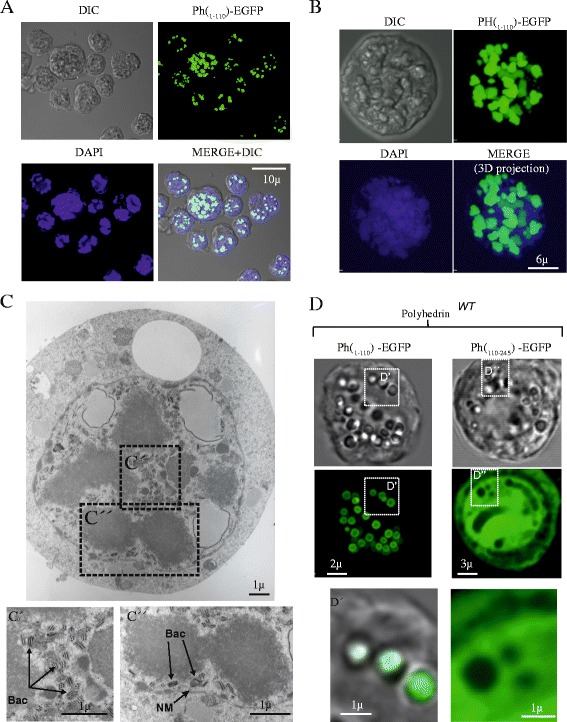
***A wild type copy of polyhedrin is required to incorporate polyhedrin fragments into the crystal*** **. A**, confocal microscopy images illustrating that the polyhedrin fragment PH_(1–110)_-EGFP forms aggregates inside the nuclei of infected cells (when expressed in the absence of WT polyhedrin). **B**, Tridimensional confocal reconstruction of a cell expressing PH_(1–110)_-EGFP. Notice the formation of aggregates inside the nucleus. **C**, The PH_(1–110)_-EGFP aggregates are visible in TEM as dense amorphous particles, and they do not contain baculoviruses inside. Notice that in fact viruses are excluded from the aggregates (C’ and C”, indicates as Bac and arrows, NM = nuclear membrane). **D**, Only the amino terminal fragment from polyhedrin can be incorporated into polyhedra crystals (when co-expressed with wild type polyhedrin). Notice that only the fragment PH_(1–110)_-EGFP form polyhedra (D’). The carboxyl terminus fragment PH_(1–110)_-EGFP is not incorporated into the polyhedra crystals, in fact it is excluded from the crystal and observed as a soluble protein in the cell cytosol (D”).

To evaluate the expression and localization of the recombinant baculoviruses containing the fused EGFP protein to different fragments of polyhedrin, we infected Sf9 cells with baculoviruses carrying the different fragments generated for this study (Figure 1A) and performed confocal and transmission electron microscopy (TEM) analysis with the cells expressing these fragments (Figure 6). The fragment PH_(1–110)_­EGFP aggregated in the nuclei (demonstrated by the co-localization with DAPI), indicating that the self-aggregation property of polyhedrin is retained in this fragment (Figure 6B). These self-aggregating structures has been previously described and named as polyhedra­like structures [4]. Similar results were obtained with the full length polyhedrin fused to EFGP (PH_(1–245)_-EGFP, Figure 4). This observation indicates that the nuclear retention signal is contained within the first 110 amino acids from polyhedrin, and that the traffic of polyhedrin to the nucleus is not affected by the fusion to EGFP. The fact that PH_(1–245)_-EGFP and PH_(1–110)_­EGFP retained the property of self-aggregation but the carboxyl terminus of polyhedrin (PH_(110–245)_­EGFP) is soluble, strengthens the hypothesis that in the fragment PH_(1–110)_­EGFP is located the sequence/structure responsible for self-aggregation.

TEM images from cells expressing PH_(1–110)_­EGFP showed electron dense intranuclear protein aggregates, corresponding to the polyhedra­like structures observed in confocal microscopy (Figure 6C). Notice that these aggregates did not contained baculoviruses; rather viruses appeared to be discarded from the aggregates (insets C’ and C”). Similar results were obtained with the full length PH_(1–245)_-EGFP recombinant polyhedrin (data not shown).

Co-expression of PH_(1–110)_­EGFP with WT polyhedrin resulted in the formation of canonical polyhedra with EGFP contained within the crystal (Figure 6D). Interestingly, in confocal images of Sf9 cells infected with the PH_(111–245_)­EGFP baculovirus, the EGFP was observed scattered throughout the cell as a soluble protein (Figure 6D). This observation further confirms that the nuclear localization signal of polyhedrin is contained within the first 110 amino acids, as previously suggested [23], and that the self-aggregating sequence from polyhedra is present within the first 110 amino acids. All these results strongly suggest that only the amino terminus from polyhedrin can be incorporated into polyhedra crystals (when co-expressed with WT polyhedrin).

To further investigate what fragments from the amino terminus of polyhedrin can be incorporated into the polyhedra, we prepared new recombinant baculoviruses containing several fragments from polyhedrin fused to EGFP.

**Figure 7:**
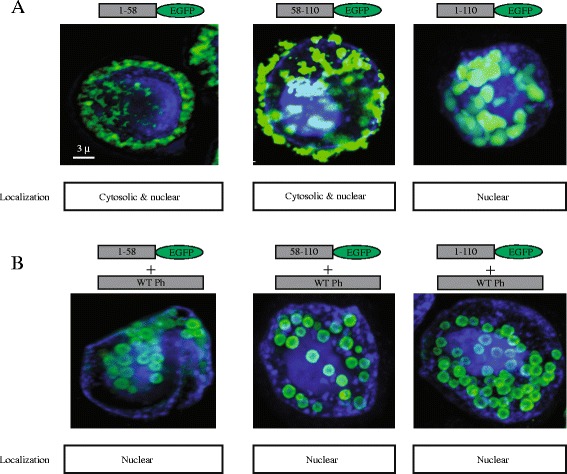
***Identification of the minimum fragment from polyhedrin that retains the self-aggregation property.*** **A**, confocal microscopy studies using the fragments PH_(1–58)_­EGFP, PH_(58–110)_­EGFP and PH_(1–110)_­EGFP alone, or in co-expression with WT polyhedrin **(B)**. Boxes below each panel indicate cellular localization based on the degree of co-localization with the nuclear marker DAPI. Notice that fragment PH_(1–58)_­EGFP is soluble and found in both the nucleus and the cytosol. Fragments PH_(58–110)_­EGFP and PH_(1–110)_­EGFP can form self-aggregates when expressed alone or be incorporated into polyhedra, when co-expressed with WT polyhedrin. Thus the minimum self-aggregating fragment identified in this study was PH_(58–110)_­EGFP.

Most interestingly, the baculoviruses expressing shorter fragment PH_(1–58)_­EGFP was observed as a soluble protein in the cytosol and nucleus of infected cells, but the fragment PH_(58–110_)­EGFP was observed as condensed, amorphous material in the nuclei and cytosol of the infected cells (Figure 7A). Co-expression with WT polyhedrin with both polyhedrin fragments resulted in canonical polyhedra formation with EGFP in the interior of the crystal (Figure 7B). These results indicate that the sequence/structure responsible for self-aggregation is in found within the 58–110 amino acids from polyhedrin, since the fragment PH_(1–58)_­EGFP is soluble and does not aggregate on its own. Nevertheless both fragments can be incorporated into canonical polyhedra when co-expressed with WT polyhedrin. The fragment PH_(1–110)_­EGFP can self-aggregate, but the aggregates are contained within the nuclei, strongly suggesting that the nuclear retention signal is found in the combination of fragments PH_(1–58)_­EGFP and PH_(58–110)_­EGFP, since separately both fragments are distributed in the nuclei and cytosol, but when combined (in the fragment PH_(1–110)_­EGFP) are exclusively present in the nuclei.

These results suggest the presence of a putative nuclear retention signal shared by fragments 1–58 and 58–110. A previous study identified a nuclear retention signal present within the sequence 1–110 [23]. However, in the aforementioned study they did not test as many polyhedrin fragments as we did in the present study.

To delimit more precisely the minimum amino acid sequence capable of forming canonical polyhedra when co-expressed with WT polyhedrin, we produced several recombinant baculoviruses carrying different fragments from the amino terminus of polyhedrin. We concentrated in the first 58 amino acids, to further delimit the minimal sequence capable of being incorporated into polyhedra.

The fragments PH_(1–25)_­EGFP and PH_(25–58)_­EGFP did not form aggregates, neither they could be incorporated into the polyhedra when co-expressed with WT polyhedrin. Similar results were obtained with the fragment PH_(1–34)_­EGFP, PH_(1–48)_­EGFP, PH_(17–58)_­EGFP or PH_(25–49)_­EGFP. These results indicate that the minimum fragment that can be incorporated into the polyhedra crystal is PH_(1–58)_­EGFP. Similarly, the fragment PH_(58–110)_­EGFP was also incorporated into the polyhedra crystal, but we did not explore other sequences within this later fragment in the present study.

All the results presented in this study show that: a) fragments from the n-terminus of polyhedrin, namely 1–58 and 58–110 can also be incorporated into the canonical polyhedra (when co-expressed with WT polyhedrin), regardless of the cellular localization or the fact that they can self-aggregate (58–110) or not (1–58); b) fragments from the c-terminus of polyhedrin are soluble, found scattered throughout the cell cytosol and nuclei, and are not incorporated into the polyhedra (when co-expressed with WT polyhedrin). In fact, they were completely excluded from the polyhedra crystal, as illustrated in Figure 6D (see also insets D’ and D”).

To obtain quantitative data about the effectiveness of the different fragments to be incorporated into the polyhedra crystal, we conducted flow cytometry studies with polyhedra formed by the combination of the different fragments from polyhedrin fused to EGFP and WT polyhedrin. Polyhedra were purified for these studies prior to conducting the flow cytometry studies (Methods).

Figure 2 shows the results of measuring EGFP fluorescence as reporter of the amount of fragments incorporated into the polyhedra crystals. Figure 2A illustrates typical histograms of single particle fluorescence and Figure 2B summarizes the results (mean ± SD) from at least 3 independent measurements. As illustrated in the figure only fragments PH_(1–58)_­EGFP, PH_(58–110)_­EGFP, PH_(1–110)_­EGFP and the full length PH_(1–245)_-EGFP were incorporated into polyhedra crystals. Most interestingly, the most fluorescent polyhedra was obtained with the fragment PH_(58–110)_­EGFP, followed by PH_(1–110)_­EGFP and the full length PH_(1–245)_-EGFP. These results strongly suggest that using the fragment PH_(58–110)_­EGFP results in higher yields of recombinant protein incorporated into the polyhedra crystals.

As we have previously shown, both PH_(1–110)_­EGFP and PH_(58–110)_­EGFP produced electron dense particles identifiable by TEM. Interestingly, both nanoparticles can be isolated from Sf9 cell lysates and retain its structural features. Nanoparticles produced by fragment PH_(1–110)_­EGFP inside Sf9 infected cells are illustrated in Figure 3A. These nanoparticles produced by the self-aggregating PH_(1–110)_­EGFP can be isolated from Sf9 lysates, as illustrated in the electron scanning images from Figure 3B. The nanoparticles have integrated EGFP and the fluorescence is observed by confocal microscopy (Figure 3C). Using an alternative method for nanoparticle size analysis based on Nanoparticle Tracking Analysis (NTA) we identified that both PH_(1–110)_­EGFP and PH_(58–110)_­EGFP produced nanoparticles of about 100 nm in diameter (6D). Notice that the main nanoparticles identified are multiples of 100 nm, which was the most abundant and smallest size identified (6D).

Figure 1A summarizes the fragments that can be incorporated into polyhedra, when co-expressed with WT polyhedrin. Figure 1B illustrates the secondary structure of polyhedrin, obtained from the crystal [10]. As indicated in this figure, polyhedrin is formed of several consecutive β sheets and α helices. The crystallographic study of polyhedrin has identified the first helix (H1) as relevant for the formation of the polyhedrin trimer, which in turn forms the basic cell of the crystal [10,11].

Our studies have identified the fragment PH_(58–110)_­EGFP as containing the sequence/structure essential for the self-aggregation properties of polyhedra (Figure 1C). Using this fragment results in self-aggregated particles scattered in the nuclei and cytosol of infected cells (Figures 1C and 7).

In spite of the fact that both minimal fragments that can be incorporated into polyhedra (PH_(1–58)_­EGFP and PH_(58–110)_­EGFP) when co-expressed with WT polyhedrin have similar structural features: both are formed by two consecutive β sheets followed by a α helix (Figure 1B), only the fragment PH_(58–110)_­EGFP retains the self-aggregation property of polyhedra.

All the results gathered in this study indicate that the sequence/structure required for a fragment to be incorporated into the polyhedra crystal are present in both fragments PH_(1–58)_­EGFP and PH_(58–110)_­EGFP. For this reason the fragment PH_(1–110)_­EGFP is also incorporated into polyhedra when co-expressed with WT polyhedrin. The fragment PH_(111–245)_­EGFP from the C-terminal region of polyhedrin was excluded from the polyhedra crystals (Figure 6D). Noteworthy, not all fragments were incorporated into polyhedra with the same efficiency, the most effective appeared to be PH_(58–110)_­EGFP (Figure 2B). This strongly suggest that the property that facilitates association to WT polyhedrin to form the crystal is in both fragments (PH_(1–58)_­EGFP and PH_(58–110)_­EGFP), but the self-aggregation property is present exclusively in fragment PH_(58–110)_­EGFP. The sequence/structure required for nuclear localization appear to be a combination of fragments PH_(1–58)_­EGFP and PH_(58–110)_­EGFP, since only the fragment PH_(1–110)_­EGFP showed exclusive nuclear localization.

## Discussion

The baculovirus expression system has become a powerful tool for recombinant eukaryotic gene expression [24]. The initial production of recombinant proteins, directed under the strong promoter of polyhedrin, was carried out in cultured cells as soluble proteins. Several modifications have been made over the last ten years to improve the system in order to obtain larger protein yields or adequate the recombinant protein for mammalian usage [25]. Some of these improvements include enhanced trafficking, folding and glycosilation, as well as preventing intracellular degradation. Another recently developed strategy has been the expression of foreign proteins incorporated into the polyhedra crystal [26]. Several proteins have been now incorporated into polyhedra, by fusing them to the full length polyhedrin in three different insect viruses [27].

Jarvis et al. fused different fragments of AcMNPV polyhedrin to two nonnuclear reporter proteins, β-galactosidase and β-glucorinadase to define its possible nuclear localization signal, and study the distribution and assembly of polyhedra by indirect immunofluorescence and by biochemical fractionation and SDS-PAGE of infected Sf9 [23]. The recombinant proteins produced diffuse or occlusion-like (self-aggregates) proteins, but none of them assembled into the polyhedra.

More recently, Je et al. using GFP fused to AcMNPV polyhedrin, observed the GFP in a soluble form in the nucleus and cytoplasm of infected cells [8]. Only when co-expressed with WT polyhedrin resulted in the formation of polyhedra. Using the elucidated crystal structure of AcMNPV polyhedra we constructed several baculovirus expressing the fluorescent protein EGFP fused to AcMNPV polyhedrin or to its different fragments in order to follow the formation of recombinant polyhedra by confocal microscopy or TEM in Sf9 infected cells.

The N-terminal from 1–110 amino acids of AcMNPV polyhedrin can be divided into two similar fragments (1–57 and 58–110) each of them consisting of two β helices (β_A_, β_A’_, and β_A”,_ β_B_ ), followed by a short α helix (α_1_ and α_2_) Figure 1B and [10].

Because the 110 amino acids N-terminal region contributes to the clamping of the 3 molecules of polyhedrin in the trimer (the core of the crystal, [10]), we considered that as the fragment 1–110 of AcMNPV, these two smaller fragments (1–58 and 58–110) could associate and direct the incorporation of foreign proteins into polyhedra. Analyzing EGFP expression through confocal microscopy and TEM, allowed us to determine the localization and formation of polyhedra in infected Sf9 cells with different polyhedrin fragments.

We observed that the fragments PH_(1–58)_, PH_(58–110)_ and PH_(1–110)_ of AcMNPV can be incorporated into polyhedra, only when co-expressed with the WT polyhedrin. The fact that polyhedra located in the nucleus is observed only when co-expressing WT and recombinant fragments of polyhedrin, suggest that the WT and the different N-terminal fragments identified in this study associate in the cytosol and travel, assembled with WT polyhedrin, to the nucleus of the infected cell. In agreement with this hypothesis, a previous study demonstrated that the nuclear localization of polyhedrin becomes more evident during the phase of occlusion due to the high rate of polyhedrin localization signal and a higher rate of protein biosynthesis [28].

An important finding in the formation of polyhedra was the ratio of the recombinant fused polyhedrin fragments to the WT polyhedrin. Because the minimal unit of polyhedra crystals is a trimer, the association of the recombinant protein could be carried out in a stoichiometric ratio of 3 WT versus 1 recombinant polyhedrin (Figure 5A), and this parameter could be very important for the formation and quantity of foreign protein that could be incorporated into the crystal.

Noteworthy, not all fragments that incorporate into polyhedra result in equal amounts of fluorescence emitted by the EGFP incorporated into the particles. Most notably the fragment PH_(58–110)_­EGFP produced the brightest particles in our study, as assayed by flow cytometry, followed by fragments PH_(1–110)_­EGFP and PH_(1–58)_­EGFP, in that order. Most surprisingly, the less efficient was the full length polyhedrin (PH_(1–245)_-EGFP, Figure 2B). Because the brightness of EGFP may reflect also geometric properties of the crystals formed by the different polyhedrin fragments, we conducted western blot analysis of the purified polyhedra formed by the combination of WT polyhedrin and the fragments presented in this study. In general agreement with the flow cytometry results, western blot analysis indicated different ratios of recombinant and WT polyhedrin present in the polyhedra particles (data not shown). These results strongly suggest a different incorporation efficiency for the different fragments studied here, pointing to PH_(58–110)_­EGFP as the most efficient of all. The reasons behind the increased incorporation of PH_(58–110)_­EGFP remains unsolved but are a target of a current study. Most interestingly, this was the minimum fragment that could form self-aggregates, thus a hint about the greater efficiency of incorporation into polyhedra may reside here.

The identification of the amino acids 58–110 as the smallest fragment capable of self-aggregation and efficient incorporation into polyhedra crystals from the N-terminal of AcMNPV is important in the development of new technologies to produce nanoparticles of interest in science and biotechnology, carrying foreign proteins of interest. Most interestingly, the self-aggregates produced by fragments PH_(1–110)_ and PH_(58–110)_ are electron dense particles, as identified in our TEM studies (Figures 3A and 6C), and they could be purified by low speed centrifugation as nanoparticles (Figure 3B-C). These later findings point to the use of fragment PH_(58–110)_ as a powerful tool to produce high yields of recombinant protein that can be easily isolated by slow speed centrifugation from Sf9 cell lysates.

## Conclusions

Using a deletion strategy based on the crystallographic structure of AcMNPV polyhedrin protein, we have identified a minimum sequence (PH_(58–110)_) consisting of two beta domains followed by an alpha helix, which contains a self aggregating domain essential for polyhedra-like particle formation. This deletion strategy allowed also the identification of a nuclear retention signal in polyhedrin, contained within the first 110 amino acids. Even though the fragment PH_(1–58)_ contains also two beta domains followed by an alpha helix, is not sufficient for self-aggregation, since the expression of this fragment results a soluble form contained in the cytosol of infected cells.

These findings open new avenues to explore how polyhedra crystals are formed, and to understand what structural features may be required for *in vivo* protein crystallization.

## References

[1] Elias CB, Jardin B, Kamen A (2007). Recombinant protein production in large-scale agitated bioreactors using the baculovirus expression vector system. Methods Mol Biol.

[2] Trowitzsch S, Bieniossek C, Nie Y, Garzoni F, Berger I (2010). New baculovirus expression tools for recombinant protein complex production. J Struct Biol.

[3] Patterson RM, Selkirk JK, Merrick BA (1995). Baculovirus and insect cell gene expression: review of baculovirus biotechnology. Env Heal Perspect.

[4] Carstens EB, Krebs A, Gallerneault CE (1986). Identification of an amino acid essential to the normal assembly of Autographa californica nuclear polyhedrosis virus polyhedra. J Virol.

[5] Ramoska WA, Stairs GR, Hink WF (1975). Ultraviolet light activation of insect nuclear polyhedrosis virus. Nature.

[6] Matsuura Y, Possee RD, Overton HA, Bishop DH (1987). Baculovirus expression vectors: the requirements for high level expression of proteins, including glycoproteins. J Gen Virol.

[7] Ooi BG, Miller LK (1990). Transcription of the baculovirus polyhedrin gene reduces the levels of an antisense transcript initiated downstream. J Virol.

[8] Je YH, Jin BR, Park HW, Roh JY, Chang JH, Seo SJ (2003). Baculovirus expression vectors that incorporate the foreign protein into viral occlusion bodies. Biotechniques.

[9] Rohrmann GF (1986). Polyhedrin structure. J Gen Virol.

[10] Ji X, Sutton G, Evans G, Axford D, Owen R, Stuart DI (2010). How baculovirus polyhedra fit square pegs into round holes to robustly package viruses. EMBO J.

[11] Coulibaly F, Chiu E, Ikeda K, Gutmann S, Haebel PW, Schulze-Briese C (2007). The molecular organization of cypovirus polyhedra. Nature.

[12] Chiu E, Coulibaly F, Metcalf P (2012). Insect virus polyhedra, infectious protein crystals that contain virus particles. Curr Opin Struct Biol.

[13] Ijiri H, Coulibaly F, Nishimura G, Nakai D, Chiu E, Takenaka C (2009). Structure-based targeting of bioactive proteins into cypovirus polyhedra and application to immobilized cytokines for mammalian cell culture. Biomaterials.

[14] Nishishita N, Ijiri H, Takenaka C, Kobayashi K, Goto K, Kotani E (2011). The use of leukemia inhibitory factor immobilized on virus-derived polyhedra to support the proliferation of mouse embryonic and induced pluripotent stem cells. Biomaterials.

[15] Matsumoto G, Ueda T, Shimoyama J, Ijiri H, Omi Y, Yube H (2012). Bone regeneration by polyhedral microcrystals from silkworm virus. Sci Rep.

[16] Furuta T, Ogawa T, Katsuda T, Fujii I, Yamaji H (2010). Efficient production of an antibody Fab fragment using the baculovirus-insect cell system. J Biosci Bioeng.

[17] Lee KS, Sohn MR, Kim BY, Choo YM, Woo SD, Yoo SS (2012). Production of classical swine fever virus envelope glycoprotein E2 as recombinant polyhedra in baculovirus-infected silkworm larvae. Mol Biotechnol.

[18] Luz-Madrigal A, Asanov A, Camacho-Zarco AR, Sampieri A, Vaca L (2013). A cholesterol recognition amino acid consensus domain in GP64 fusion protein facilitates anchoring of baculovirus to mammalian cells. J Virol.

[19] Wright M (2012). Nanoparticle tracking analysis for the multiparameter characterization and counting of nanoparticle suspensions. Methods Mol Biol.

[20] Filipe V, Hawe A, Jiskoot W (2010). Critical evaluation of Nanoparticle Tracking Analysis (NTA) by NanoSight for the measurement of nanoparticles and protein aggregates. Pharm Res.

[21] Xiang X, Yang R, Chen L, Hu X, Yu S, Cao C (2012). Immobilization of foreign protein into polyhedra of Bombyx mori nucleopolyhedrovirus (BmNPV). J Zhejiang Univ Sci B.

[22] Chen L, Xiang X, Yang R, Hu X, Cao C, Malik FA (2013). Immobilization of foreign protein in BmNPV polyhedra by fusion expression with partial polyhedrin fragments. J Virol Methods.

[23] Jarvis DL, Bohlmeyer DA, Garcia A (1991). Requirements for nuclear localization and supramolecular assembly of a baculovirus polyhedrin protein. Virology.

[24] Wang KC, Wu JC, Chung YC, Ho YC, Chang MD, Hu YC (2005). Baculovirus as a highly efficient gene delivery vector for the expression of hepatitis delta virus antigens in mammalian cells. Biotechnol Bioeng.

[25] Hitchman RB, Possee RD, King LA (2009). Baculovirus expression systems for recombinant protein production in insect cells. Recent Pat Biotechnol.

[26] Ikeda K, Nakazawa H, Shimo-Oka A, Ishio K, Miyata S, Hosokawa Y (2006). Immobilization of diverse foreign proteins in viral polyhedra and potential application for protein microarrays. Proteomics.

[27] Coulibaly F, Chiu E, Gutmann S, Rajendran C, Haebel PW, Ikeda K (2009). The atomic structure of baculovirus polyhedra reveals the independent emergence of infectious crystals in DNA and RNA viruses. Proc Natl Acad Sci U S A.

[28] Jarvis DL, Bohlmeyer DA, Garcia A (1992). Enhancement of polyhedrin nuclear localization during baculovirus infection. J Virol.

